# Whole Transcriptome Sequencing of Peripheral Blood Shows That Immunity/GnRH/PI3K-Akt Pathways Are Associated With Opioid Use Disorder

**DOI:** 10.3389/fpsyt.2022.893303

**Published:** 2022-06-21

**Authors:** Qi Dai, Shan-Shan Pu, Xue Yang, Chang Li, Yafei He, Xiaobo Liu, Gang Wang

**Affiliations:** ^1^Affiliated Wuhan Mental Health Center, Jianghan University, Wuhan, China; ^2^School of Mental Health and Psychological Sciences, Anhui Medical University, Hefei, China; ^3^Department of Addiction, Wuhan Mental Health Center, Wuhan, China; ^4^Department of Psychiatry, Wuhan Mental Health Center, Wuhan, China

**Keywords:** OUD, RNA-seq, lncRNA, immunity, GnRH secretion, PI3K-Akt signaling pathway

## Abstract

**Background:**

Opioid use disorder (OUD), which is most commonly exhibited as addiction, is a persistent chronic disease that places a burden on families and society. Various peripheral traits have been linked to OUD in the past, but research on this topic is insufficient.

**Methods:**

Seven male patients with OUD and 7 male healthy controls with matched demographic and clinical data were enrolled in this study. Peripheral blood RNA was used to construct an rRNA-removed library and a small RNA library. The peripheral transcriptomic differences between the two groups were investigated using RNA-seq. Differentially expressed messenger RNAs (mRNAs), long non-coding RNAs (lncRNAs), circular RNAs (circRNAs) and microRNAs (miRNAs) were identified by bioinformatics methods, and functional enrichment analysis with differentially expressed RNAs was performed to investigate the potential biological mechanisms of OUD.

**Results:**

A total of 229 mRNAs (115 upregulated, 114 downregulated), 416 lncRNAs (191 upregulated, 225 downregulated), 17 circRNAs (16 upregulated, 1 downregulated) and 74 miRNAs (42 upregulated, 32 downregulated) were differentially expressed between the OUD group and the healthy control group. Functional enrichment analysis with differentially expressed mRNAs showed that immunity, GnRH secretion, and PI3K-Akt signaling pathways were associated with OUD. Immunity-, JAK-STAT-, and insulin-related pathways were enriched in functional enrichment analysis of target genes predicted by differentially expressed miRNAs.

**Conclusion:**

We identified hundreds of differentially expressed genes that were enriched in immunity, GnRH secretion and PI3K-Akt signaling pathways. Some genes with significant changes might be used as potential biomarkers for progression and treatment of OUD.

## Introduction

Opioid use disorder (OUD) is a chronic relapsing disorder characterized by loss of control of opioid drugs use, compulsive use, and continued use despite harm (1). These drugs include prescription painkillers such as morphine and illegal drugs such as heroin. According to an estimate by the Global Burden of Disease study in 2016, there were 26.8 million people with OUD worldwide (2). Opioid use outside of its appropriate clinical applications can directly cause physical harm and potential health sequelae, such as virus infection due to shared syringes. In addition, opioid abuse is associated with wider societal costs, a high divorce rate, reduced employment and a high crime rate (3). The physical and social harms caused by the abuse of opioids have become an increasingly serious public health issue.

Multiple brain regions, such as the nucleus accumbens (NAc), central nucleus of the amygdala (CeA) and prefrontal cortex (PFC), as well as the peripheral system, have been reported to be associated with addiction (3). Previous studies have revealed that metabolism, endocrine systems, immune systems and mitochondria play important roles in the process of long-lived behavioral abnormalities associated with addiction (4–8). Opiate addiction leads to autophagy-mediated dysfunction of mitochondria, such as a decrease in mitochondrial DNA (mtDNA) copy number in the hippocampus and peripheral blood and an increase in reactive oxygen species (ROS), which contributes to cell damage and apoptosis (6). In addition, recent studies have suggested that many cytokines and regulatory T cells in peripheral blood were dysregulated in patients with heroin addiction compared to healthy controls (8–11). Women with addiction had lower oxytocin levels in their peripheral blood, which could be used as a biomarker for predicting the intensity of social anxiety in female patients with heroin withdrawal (12). Overall, although there are many studies on the peripheral blood characteristics of addiction, fewer studies have focused on the systematic peripheral changes in OUD.

With the advances of high-throughput sequencing, we can now systematically investigate the transcriptome profile, including mRNAs, long non-coding RNAs (lncRNAs), circular RNAs (circRNAs) and miRNAs, in peripheral blood. In the present study, we enrolled 7 patients with OUD and 7 healthy controls and analyzed the transcriptome expression of peripheral blood by RNA sequencing. We identified hundreds of differentially expressed transcripts (mRNAs, lncRNAs, circRNAs, and miRNAs), and enrichment analysis with these differentially expressed transcripts suggested that several pathways might participate in the mechanisms of OUD.

## Materials and Methods

### Participants and Ethics Statement

A total of 14 male participants, 7 patients with OUD and 7 healthy control subjects aged 40–50 years, who met the Diagnostic and Statistical Manual of Mental Disorders, 5th Edition (DSM-5), diagnostic criteria for Opioid Use Disorder 1 (Supplementary Table S1). A problematic pattern of opioid use leading to clinically significant impairment or distress, as manifested by at least two of the following, occurring within a 12-month period: Opioids are often taken in larger amounts or over a longer period than was intended. All of them had nicotine dependence, were recruited from Wuhan Mental Health Center, Wuhan, China. We excluded subjects with the following criteria: (1) the subjects had polydrug use; (2) the subjects had a serious physical illness; (3) subjects with primary mental illness in the OUD group and those with mental illness in the control group. All participants provided written informed consent before enrollment. The protocols and recruitment procedures described in the present study were approved by the Research Ethics Committee of Wuhan Mental Health Center (Ky2022.02.04). The history of heroin use and current medication use was obtained via self-report and electronic medical records. Five milliliters of blood was drawn from each of the participants with an empty stomach at the same time of day in the morning.

### Library Construction and Transcriptome Sequencing

Total RNA was extracted from the blood using the PAXgene^®^ Blood RNA Kit (Qiagen, Hombrechtikon, Switzerland) according to the handbook's instructions. The purity, concentration and integrity of total RNA were assessed using a NanoPhotometer^®^ spectrophotometer (Implen, CA, USA), Qubit^®^ RNA Assay Kit with a Qubit^®^ 2.0 Fluorometer (Life Technologies, CA, USA) and RNA Nano 6,000 Assay Kit of the Agilent Bioanalyzer 2,100 system (Agilent Technologies, CA, USA), respectively. In addition, RNA degradation and contamination were monitored on 1% agarose gels. To get the overview of whole transcriptome including the long transcripts (mRNAs, lncRNAs and circRNAs) and small RNAs (miRANs), the rRNA-removed library and small RNA library were constructed separately. For the rRNA-removed library, a total amount of 3 μg RNA per sample was used as input material for the RNA sample preparations. Ribosomal RNA was removed by an Epicenter Ribo-zeroTM rRNA Removal Kit (Epicenter, USA). Sequencing libraries were generated using the NEBNext^®^ UltraTM RNA Library Prep Kit for Illumina^®^ (NEB, USA) following the manufacturer's recommendations, and index codes were added to attribute sequences to each sample. The library preparations were sequenced on an Illumina HiSeq platform to generate 150 bp paired-end reads. For the small RNA library, a total of 2 μg of total RNA was isolated from each sample using the NEBNext^®^ Multiplex Small RNA Library Prep Set For Illumina^®^ (NEB, USA) according to the manufacturer's instructions. Small RNA library preparations were sequenced on a NovaSeq 6,000 platform, and 50 bp single-end reads were generated.

### RNA-Seq Data Processing

For rRNA-removed library sequencing data, the raw sequencing reads were first processed to remove sequencing adapters and low-quality reads using Trimmomatic (version 0.39) with default parameters (13). For mRNA and lncRNA quantification, the clean reads were mapped to the human reference genome GRCh38 using HISAT2 (version 2.2.1) (14) and then sorted by samtools (version 0.11.0) (15) with the sort function by genome position. FeatureCounts (version 2.0.1) (16) was used to count reads mapped to specific meta-features (mRNA or lncRNA). We downloaded the genome reference data and gene annotation file (Release 35) from the GENCODE website (https://www.gencodegenes.org/) (17). Since the gene annotation file from GENCODE contained only some lncRNAs, we downloaded the lncRNA annotation file from the NONCODE database (http://www.noncode.org/) (18, 19) and combined all the lncRNAs from the two databases. In total, 173,112 transcripts from 96,409 lncRNA genes were analyzed in this study. We quantified circRNA expression using the CIRIquant (version 1.1.1) process (https://ciri-cookbook.readthedocs.io/en/latest/CIRIquant_0_home.html) (20). Briefly, the clean reads were first mapped to the human reference genome using bwa (version 0.7.17) (21). The backsplice junction reads were identified and quantified by CIRI2 (version 2.0.6) (22, 23) and CIRIquant (20). For small RNA analysis, the miRNAs were quantified by miRDeep2 (24) using human miRNAs from miRbase (25) as a reference.

### Differential Expression and Functional Enrichment

We merged all the mRNA, lncRNA and circRNA count files into one file because they were sequenced in one library. The DESeq2 (26) R package was used to conduct the differential expression analysis (mRNAs, lncRNAs, circRNAs and miRNAs). DEseq2 first fitted the reads count with negative binomial distribution model, and then conducted differential expression analysis by Ward test, which used the estimated standard error of a log_2_fold change to test if it is equal to zero. We used the multifactor design formula “design = ~age + height + weight + yearofedu + group” in DESeq2 to rule out the effect of confounding factors, including age, height, weight and years of education. Differentially expressed RNAs were defined with the following criteria: upregulated or downregulated 1.5 times and *p*-value < 0.01. Gene set enrichment analysis (GSEA) was used to conduct Gene Ontology (GO) and Kyoto Encyclopedia of Genes and Genomes (KEGG) enrichment analyses with the ClusterProfiler (27) R package. Functional enrichment of genes targeted by differentially expressed miRNAs was conducted on the miEAA 2.0 website (28, 29). miEAA is a web-based application that provides miRNA function enrichment by their targeted genes.

### Protein-Protein Interaction and Network Analysis

All differentially expressed mRNAs were used to construct a PPI network by STRING V11.5 (30, 31). To improve the network quality, we set the minimum required interaction score as high confidence (0.7). To construct the coexpression network, we selected the differentially expressed genes from the PI3K-Akt signaling pathway and GnRH secretion pathway and calculated the Pearson correlation coefficient of these genes with other RNAs (mRNA, lncRNA, circRNAs and miRNAs). Only the gene pairs with Pearson correlation coefficients >0.85 or < -0.85 were considered to be coexpressed. We also selected differentially expressed miRNAs and mRNAs to construct the network of miRNA-targeted mRNAs by Watson Crick pairing of nucleotides. miRWalk 2.0 was used to predict the differentially expressed miRNA-targeted mRNAs (32). To improve the prediction accuracy, we reserved genes that were also predicted by TargetScan (33). Because there were too many predicted target genes, we only showed the differentially expressed genes targeted by differentially expressed miRNAs. All the networks are displayed with Cytoscape (34).

## Results

### Hundreds of Genes Were Differentially Expressed Between Patients With OUD and Healthy Controls

**Figure 1:**
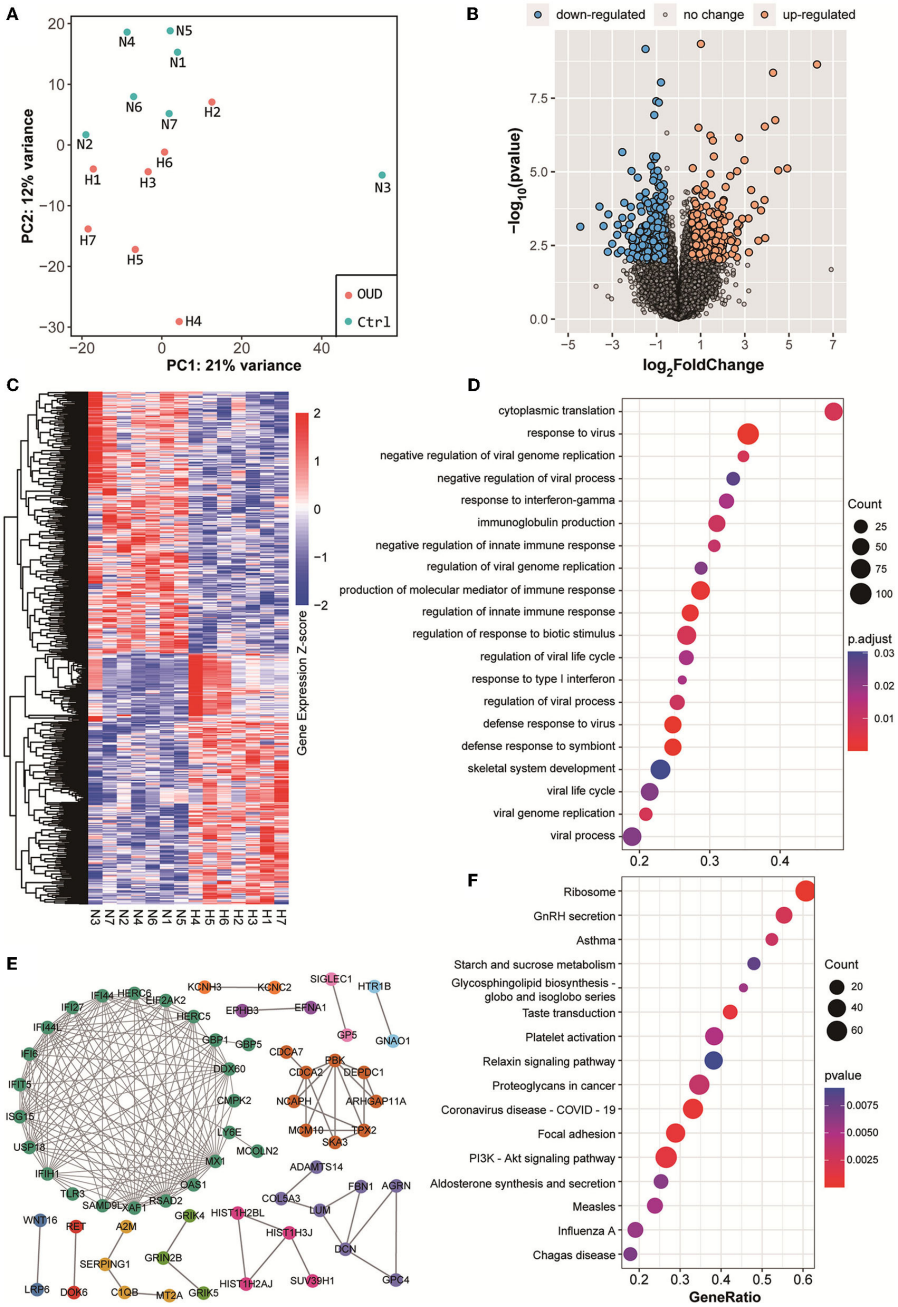
Results of rRNA removed RNA-seq. **(A)** Principal component analysis results of all samples. The red dot in the figure represented the OUD group and blue dot represented the control group. **(B)** Volcano plot of all expressed genes. We set the threshold of differential expression as |log_2_FoldChange| > 1.5 & *p*-value < 0.01. The blue dot, orange dot and gray dot represented the down-regulated genes (OUD/Control), up-regulated genes and non-significant changed genes, respectively. **(C)** Expression heatmap of all differentially expressed RNAs, including mRNA, lncRNAs and circRNAs. **(D)** GO biological process enrichment analysis results with differentially expressed mRNAs. **(E)** Protein-protein interaction results of differentially expressed genes. Gene names rendered in different colors indicated that they were in different protein interaction networks. **(F)** KEGG pathway enrichment analysis results with differentially expressed mRNAs.

**Figure 2:**
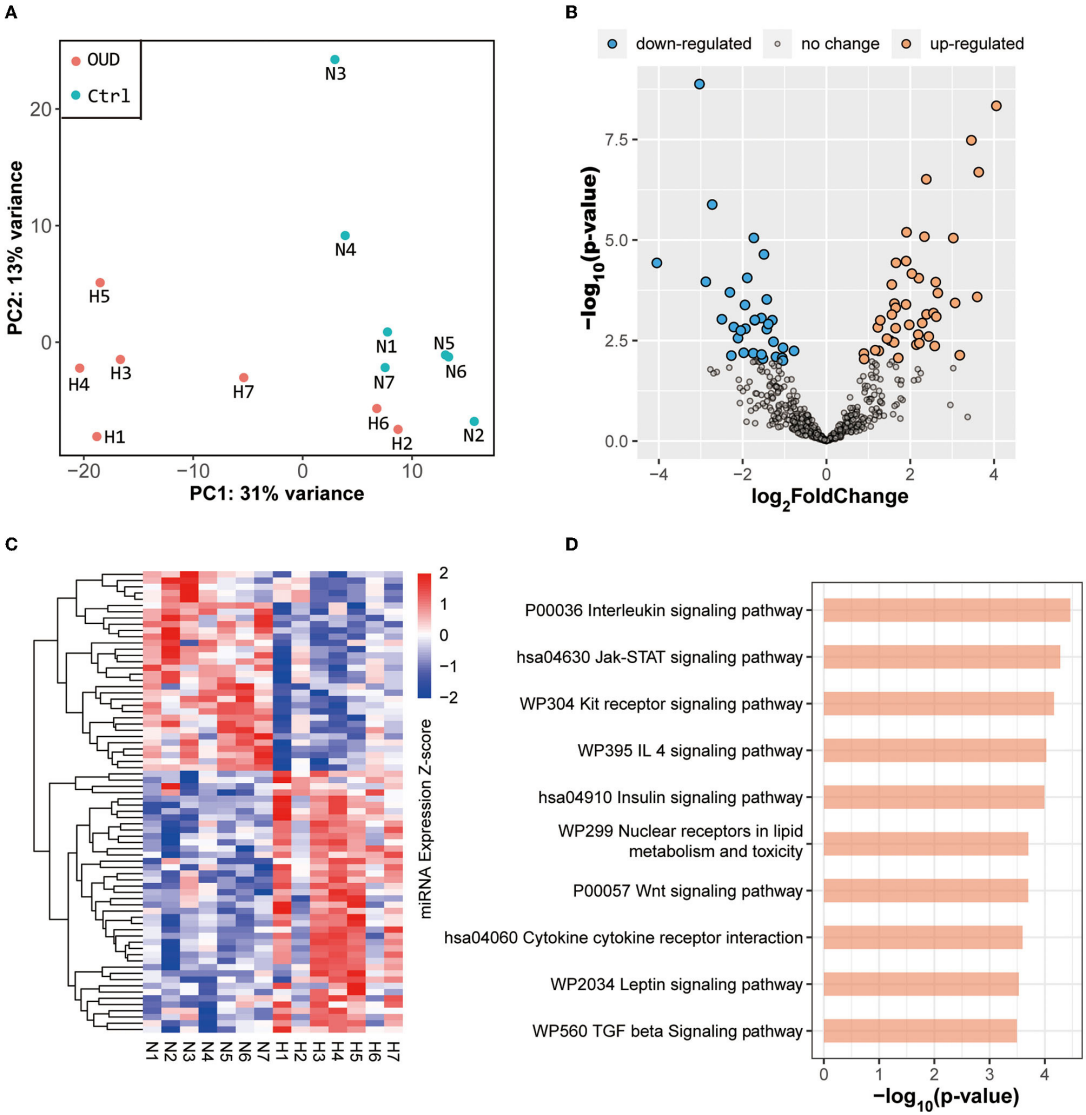
Results of small RNA sequencing. **(A)** Principal component analysis results of all samples. The red dot in the figure represented the OUD group and blue dot represented the control group. **(B)** Volcano plot of all expressed miRNAs. We set the threshold of differential expression as |log_2_FoldChange| > 1.5 & *p*-value < 0.01. The blue dot, orange dot and gray dot represented the down-regulated miRNAs (OUD/Control), up-regulated miRNAs and non-significant changed miRNAs, respectively. **(C)** Expression heatmap of all differentially expressed miRNAs. **(D)** KEGG enrichment result of targeted genes predicted by differentially expressed miRNAs.

We obtained whole transcriptome data from 7 patients with OUD and 7 healthy control subjects. The raw sequencing data of every sample from rRNA-removed sequencing and small RNA sequencing were more than 13 G (bases) and 10 M (reads), respectively. The OUD group and healthy control group were distinct, according to the results of principal component analysis (Figures 1A, 2A). A total of 229 mRNAs (115 upregulated, 114 downregulated), 416 lncRNAs (191 upregulated, 225 downregulated), 17 circRNAs (16 upregulated, 1 downregulated) and 74 miRNAs (42 upregulated, 32 downregulated) were differentially expressed between the OUD group and the healthy control group (Figures 1B,C, 2B,C; Supplementary Tables S2, S3).

### Differentially Expressed Genes Were Enriched in the Immune System and PI3K-Akt Signaling Pathway

Next, we conducted GO and KEGG gene function enrichment analyses with the differentially expressed genes. Many immune-related biological processes were enriched as a result of GO enrichment, including the response to virus (enrichment score = 0.47, *p* value = 5.24 × 10^−8^) and the production of molecular mediators of the immune response (enrichment score = 0.46, *p*-value = 1.58 × 10^−6^) (Figure 1D). GnRH (gonadotropin-releasing hormone) secretion (enrichment score = −0.54, *p* value = 2.37 × 10^−3^), taste transduction (enrichment score = −0.61, *p*-value = 9.68 × 10^−4^), and the PI3K-Akt signaling pathway (enrichment score = −0.40, *p*-value = 1.06 × 10^−3^) were significantly enriched according to KEGG enrichment results (Figure 1F). These results suggested that the immune process was positively associated with OUD, whereas GnRH secretion and the PI3K-Akt signaling pathways were negatively associated with OUD.

### PPI Network and Coexpression Network

**Figure 3:**
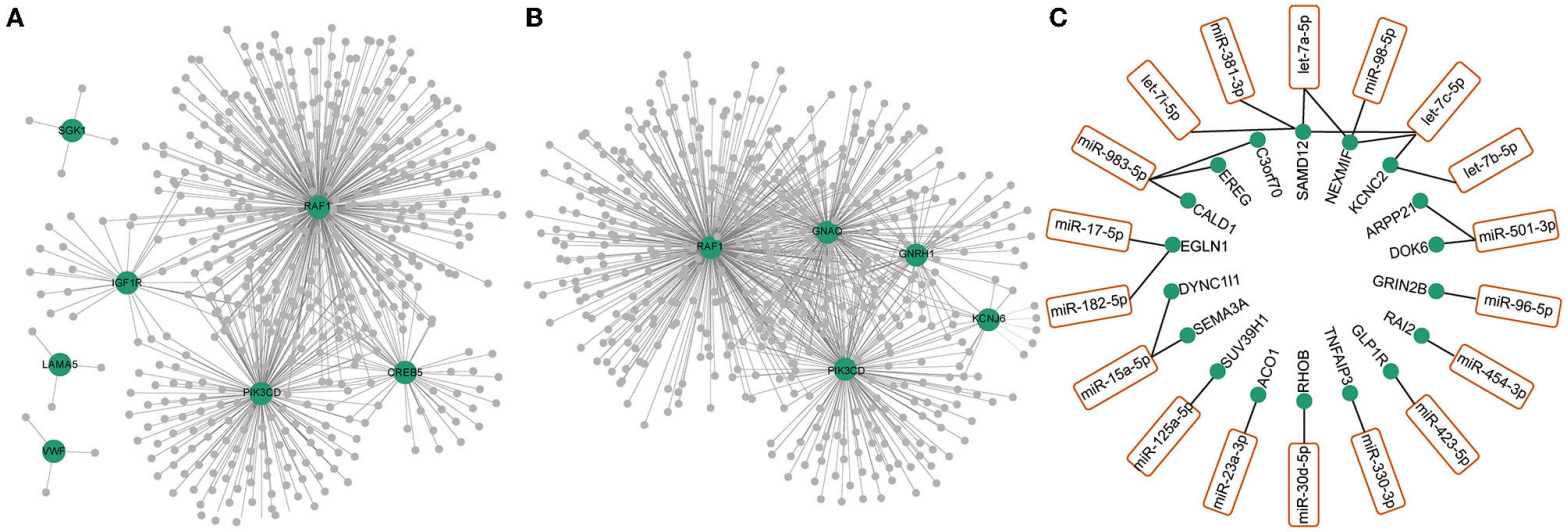
Co-expression network of differentially expressed RNAs (mRNAs, lncRNAs, circRNAs and miRNAs) with differentially expressed RNAs in GnRH secretion **(A)** and PI3K-Akt signaling pathway **(B)**. The green dots represented the differentially expressed mRNAs in GnRH secretion **(A)** and PI3K-Akt signaling pathway **(B)**. The gray dots represented the differentially expressed ncRNAs (lncRNAs, circRNAs, miRNAs) coexpressed with differentially expressed mRNAs in these two pathways. **(C)** Differentially expressed miRNAs (orange box) and their targeted mRNAs (green dots).

Differentially expressed genes constituted a large PPI network and several small PPI networks (Figure 1E). The proteins in the largest network, such as IFIT5, ISG15, IFIH1, and OAS1, were mostly associated with immunity. A smaller network, which included MCM10, CDCA2, and PBK, was linked to DNA replication and DNA stability. In particular, we found that three proteins, GRIK4, GRIN2B, and GRIK5, which encode subunits of glutamate receptors, could interact with each other. The genes differentially expressed in the PI3K-Akt signaling (Figure 3A) and GnRH secretion pathways (Figure 3B) were found to be coexpressed with many additional differentially expressed mRNAs and ncRNAs in the coexpressed network. A number of differentially expressed mRNAs were targeted by differentially expressed miRNAs (Figure 3C). Some of these differentially expressed mRNAs were involved in immunity or PI3K-Akt signaling pathways, such as *EREG, TNFAIP3* and *RHOB*. In addition, we noted that some of these genes were associated with brain function or related disorders, such as neuronal development (*C3orf70, DOK6, GRIN2B*), cognitive or intellectual disorder (*RAI2, ARPP21*), schizophrenia and Alzheimer's disease (*SEMA3A*), and epilepsy (*NEXMIF, KCNC2, SAMD12, GRIN2B*). These results further suggested that the immune system, PI3K-Akt signaling and GnRH secretion pathways might be related to opioid addiction. Moreover, some miRNAs played key roles in the process of addiction and led to addiction-related symptoms by working together with targeted mRNAs.

### Functional Enrichment of Genes Targeted by Differentially Expressed miRNAs

The predicted genes targeted by differentially expressed miRNAs were mainly enriched in several pathways, including the interleukin (*p*-value = 3.45 × 10^−5^), Jak-STAT (*p*-value = 5.27 × 10^−5^), insulin (*p*-value = 1.02 × 10^−4^) and Wnt (*p*-value = 2.00 × 10^−4^) signaling pathways (Figure 2D). The results of functional enrichment of differentially expressed miRNAs and differentially expressed mRNAs were partially consistent, especially in immune-related pathways. Moreover, although the Jak-STAT pathway is not directly related to immunity, the expression of cytokines, including interleukin and interferon, is regulated by Jak-STAT (35, 36). This evidence, in line with the mRNA enrichment results, further confirmed that the immune system was indeed involved in the pathogenesis of OUD.

## Discussion

In this study, we analyzed the whole transcriptome characteristics of peripheral blood samples from patients with OUD and healthy control subjects. We found that many genes in the immune system were differentially expressed. Immunity has long been reported to be associated with opioid addiction, as injection use of heroin contributes significantly to virus transmission (37, 38). This finding might be due to the sharing of injection equipment among drug users (39, 40). However, higher viral loads have also been detected in virus-infected heroin users than in infected non-heroin users (41), and opioid abuse has been proposed to undermine IFN-mediated antiviral innate immunity and enhance virus replication *in vitro* (42–44). Recent studies also suggested that heroin dependence could suppress adaptive immune responses by reducing the proliferation of regulatory T cells and the secretion of proinflammatory cytokines (9, 10). Moreover, many cytokines, including interleukin and interferon, fluctuate significantly in patients with OUD compared with healthy controls (8). All this evidence suggests that immune changes caused by opiate abuse might come from two sources: sharing injection equipment and reduction of immunity. When treating patients with OUD, it is also important to consider the patient's immunity.

We also found that opioid addiction could suppress GnRH secretion. Opioids can bind to opioid receptors in the hypothalamus, pituitary and testis to modulate gonadal function (5). Previous researchers have also shown that opioids could decrease plasma testosterone levels by suppressing hypothalamic GnRH and luteinizing hormone release (45). Moreover, the endocrine system, including growth hormone, prolactin, luteinizing hormone, testosterone, estradiol and oxytocin, has been reported to be affected by opioids through the hypothalamic-pituitary-adrenal (HPA) axis (45). In our study, we also found that the synthesis and secretion of aldosterone (enrichment score = −0.48, *p*-value = 6.44 × 10^−3^), which is an adrenocortical hormone, was related to opioid addiction. Therefore, our studies further indicated that opioids could affect the endocrine system.

The PI3K-Akt signaling pathway is associated with many human diseases and can regulate a variety of important cellular pathways, such as the mammalian target of rapamycin (mTOR), immune regulation, insulin and mitogen-activated protein kinase signaling pathways (46–50). Previous studies showed that selectively blocking the spinal dopamine D2 receptor (D2DR) could attenuate morphine tolerance in mice by inhibiting PI3K-Akt-MAPK signaling, and activation of PI3K-Akt signaling could promote the development of morphine tolerance (51, 52). In addition, electroacupuncture could delay the occurrence of morphine tolerance in rats by downregulating the protein expression of phosphorylated PI3K and phosphorylated Akt, which are two key molecules in the PI3K-Akt pathway (53). A recent study showed that the PI3K-Akt signaling pathway was activated after biphalin, a dimeric opioid peptide, and this effect could be reversed by opioid receptor inhibitors (54). Our findings that many genes in the PI3K-Akt pathway were differentially expressed, in line with previous studies, further suggested that PI3K-Akt participated in the pathogenesis of OUD and could be used as a potential therapeutic target.

In the present study, many ncRNAs were differentially expressed, suggesting that ncRNAs might be involved in the molecular pathogenesis of OUD. ncRNA has important regulatory functions and has been implicated in a variety of human diseases (55, 56). Not all differentially expressed ncRNAs were involved in the pathogenesis of OUD, and most of them might only be byproducts of OUD. However, regardless of whether these ncRNAs are directly related to OUD, ncRNAs with obvious expression changes, such as hsa-let-7i−3p (log_2_FC = −4.05, *p*-value = 3.72 × 10^−5^) and hsa-miR-151a-3p (log_2_FC = −4.06, *p*-value = 4.62 × 10^−9^), might serve as biomarkers for progression or treatment of OUD.

Our study has several limitations. First, the sample size in our study was small, as we only collected 14 samples and could not conduct weighted gene coexpression network analysis (WGCNA) (57). Although we identified hundreds of OUD-related genes, the results need to be verified in a larger cohort. Second, age, history of drinking and smoking were matched between the two groups, but clinical statistics, including duration, type and dose of drug use, were inconsistent within the OUD group. These confounders, although difficult to measure, could affect the results and should not be neglected. Third, we identified many ncRNAs that were differentially expressed, but we did not determine their possible biological functions. This study was only an exploratory study, and functional experiments are needed to verify the results in the future.

In summary, we identified hundreds of differentially expressed genes associated with OUD that were enriched in immunity, GnRH secretion and PI3K-Akt signaling pathways. The results of this study will help to further explain the pathogenesis of OUD and provide potential biomarkers for the treatment of OUD, but the findings need to be verified in studies with more samples.

## Data Availability Statement

The datasets presented in this study can be found in online repositories. The names of the repository/repositories and accession number(s) can be found below: Gene Expression Omnibus database under the accession number GSE198123.

## Ethics Statement

The studies involving human participants were reviewed and approved by Ethical Committee of Wuhan Mental Health Center. The patients/participants provided their written informed consent to participate in this study.

## Author Contributions

XL and GW designed the study. QD, S-SP, XY, CL, and YH recruited the participants and collected the blood samples. QD and S-SP analyzed and explained the data. GW drafted the manuscript. All authors contributed to manuscript revision, read, and approved the submitted version.

## Funding

The study was supported by the National Key R&D Program of China (2018YFC13114300 to GW) and Wuhan Medical Research program (WX19Y22 to XY).

## Conflict of Interest

The authors declare that the research was conducted in the absence of any commercial or financial relationships that could be construed as a potential conflict of interest.

## Publisher's Note

All claims expressed in this article are solely those of the authors and do not necessarily represent those of their affiliated organizations, or those of the publisher, the editors and the reviewers. Any product that may be evaluated in this article, or claim that may be made by its manufacturer, is not guaranteed or endorsed by the publisher.
